# Estimation of the Continuous Walking Angle of Knee and Ankle (Talocrural Joint, Subtalar Joint) of a Lower-Limb Exoskeleton Robot Using a Neural Network

**DOI:** 10.3390/s21082807

**Published:** 2021-04-16

**Authors:** Taehoon Lee, Inwoo Kim, Soo-Hong Lee

**Affiliations:** Department of Mechanical Engineering, Yonsei University, Seoul 03722, Korea; thoon13@yonsei.ac.kr (T.L.); polarmonkey@yonsei.ac.kr (I.K.)

**Keywords:** knee and ankle angle estimation, lower-limb exoskeleton, walking pattern, real-time motion profiles, artificial neural network

## Abstract

A lower-limb exoskeleton robot identifies the wearer′s walking intention and assists the walking movement through mechanical force; thus, it is important to be able to identify the wearer′s movement in real-time. Measurement of the angle of the knee and ankle can be difficult in the case of patients who cannot move the lower-limb joint properly. Therefore, in this study, the knee angle as well as the angles of the talocrural and subtalar joints of the ankle were estimated during walking by applying the neural network to two inertial measurement unit (IMU) sensors attached to the thigh and shank. First, for angle estimation, the gyroscope and accelerometer data of the IMU sensor were obtained while walking at a treadmill speed of 1 to 2.5 km/h while wearing an exoskeleton robot. The weights according to each walking speed were calculated using a neural network algorithm programmed in MATLAB software. Second, an appropriate weight was selected according to the walking speed through the IMU data, and the knee angle and the angles of the talocrural and subtalar joints of the ankle were estimated in real-time during walking through a feedforward neural network using the IMU data received in real-time. We confirmed that the angle estimation error was accurately estimated as 1.69° ± 1.43 (mean absolute error (MAE) ± standard deviation (SD)) for the knee joint, 1.29° ± 1.01 for the talocrural joint, and 0.82° ± 0.69 for the subtalar joint. Therefore, the proposed algorithm has potential for gait rehabilitation as it addresses the difficulty of estimating angles of lower extremity patients using torque and EMG sensors.

## 1. Introduction

Exoskeleton robots are worn on specific parts of the body to prevent external shocks in advance, to provide strong muscle strength and endurance, and for rehabilitation and assistance to patients. As such, they are being developed in various forms and for various uses in industrial, military, and rehabilitation fields to aid humans [1,2,3]. In addition, as the proportion of elderly people worldwide is increasing rapidly, it is expected that there will be a large number of rehabilitation patients by 2050 due to an aging society [4]. As the physical functions of the elderly deteriorate as they age, the need for wearable robots that help in daily life will increase. Exoskeleton robots are also used for rehabilitation training and walking assistance for stroke patients [5,6,7].

In the lower-limb exoskeleton robot for gait assistance, it is important to understand the wearer′s gait intention and to control the gait stably [8,9]. In gait training, appropriate gait assistance is performed through predetermined gait patterns, gait section prediction, gait pattern estimation, etc. [10,11,12]. For the gait section prediction, many studies are being conducted to identify gait intervals, toe-off points, and heel strike points using surface electromyography (sEMG) sensors, foot sensors using force sensing resistors (FSRs), and encoders [13,14,15,16,17], etc.; however, little research is being conducted on continuous gait pattern estimation.

In addition, algorithms were designed using various methods, such as fuzzy theory, Bayesian inference, adaptive neuro-fuzzy inference, human center of pressure (COP) analysis, and vision as a gait section prediction method [18,19,20].

In the case of leg patients, most of the leg muscle strength is reduced, making it difficult to measure the muscle signals through EMG and the joint rotation through encoders during walking. In the case of stroke patients without proper ankle function, it is difficult to estimate the gait section with the foot sensor and encoder due to the foot drop phenomenon. Furthermore, most lower-limb exoskeleton robots only consider the sagittal plane, which does not coincide with the actual axis of the ankle. Therefore, it can only move against the talocrural joint, which is not considered for the subtalar joint of the ankle. 

However, the subtalar joint of the ankle plays an important role in balancing the gait [21,22]. The proposed method can improve gait quality by estimating the rotational change of the subtalar joint during the gait. Therefore, in this study, we proposed a method to estimate the real-time rotation changes of knee and ankle during walking by applying a neural network to the data using a gyroscope and accelerometer of two inertial measurement unit (IMU) sensors attached to the thigh and shank. 

When analyzing the human gait section, from the toe-off section to the heel strike section, the knee advances first, and then the shank advances [23]. For this reason, if the knee angle is estimated using both the inertia information of the thigh and shank of a patient whose leg muscles are not functioning properly, the inertia information of the shank overlaps to the uncertainty of the thigh. Thus, to estimate the knee angle, only the inertia information of the thigh was used. It is difficult to estimate the ankle angle with only this inertial information. However, the inertia information of the shank reduces the uncertainty through the estimated knee angle, so the angle of the ankle was estimated using the inertia information of the thigh and shank.

As a result, the proposed method allows patients who have difficulty in moving the knee or ankle properly to estimate the knee rotation angle by moving the thigh and the angle of the two axes of the ankle by the movement of the thigh and shank.

Artificial neural networks (ANNs) are mathematical models of human biological neurons. These neurons take multiple input values and output a value when it exceeds a certain level based on the activation function [24]. Most ANNs are used for pattern recognition and classification. The proposed neural network algorithm is a method of continuously estimating the angle of the joint. It consists of a method of obtaining the weight and bias values of the neural network in advance and a method of running a feedforward neural network in real-time using the obtained weight and bias values. 

First, the method of calculating the weight and bias values is obtained using the neural network algorithm programmed in MATLAB software, and the input is the value of the three-axes of the IMU sensor′s gyroscope and accelerometer. The two IMU sensors consist of a total of 12 datapoints. Compared to the gyroscope, the accelerometer data fluctuates significantly, and thus the data are organized through a low pass filter to increase the accuracy of the neural network. The output layer was implemented with three outputs: the knee joint, talocrural joint, and subtalar joint. 

In the second step, the weight and bias values obtained through simulation are stored in the MCU memory, and the IMU data received in real-time during walking is processed through the feedforward neural network algorithm to predict the walking speed. Afterward, the angle of the lower-limb joint is estimated by performing the feedforward neural network process again with appropriate weight and bias values suitable for the walking speed. By using this method to secure the weight value in advance, it is possible to reduce the number of operations of the microcontroller unit (MCU). In addition, since the weight of the neural network according to the walking speed can be obtained, the accuracy in angle estimation is improved, and the angle tracking error is reduced.

To identify the toe-off point and the heel strike point of walking, the section can be estimated using only the data of the IMU sensor by comparing the foot sensor and the IMU sensor. 

In general, gait rehabilitation training with the help of an exoskeleton robot is performed at a constant walking speed. However, if the walking speed changes during gait training, the angle estimation may be less accurate. Therefore, accurate angle estimation must be performed according to the varying walking speed. For this reason, three experiments were conducted. The first experiment was to estimate the angle of the joint for each constant walking speed, the second experiment was to estimate the angle of the joint when the speed changes, and the third experiment was free walking. These three experiments showed that the proposed algorithm estimated the joint angle well.

The remainder of this paper is structured as follows. In Section 2.1, we describe the designed exoskeleton robot and walking data. In Section 2.2, we explain the gait section estimation. In Section 2.3, we describe the weight learning and data classification. In Section 2.4, simulations for the analyzed weights are presented. In Section 2.5, we present the process of estimating a joint angle in real-time. In Section 3, the experiment is organized, and the experimental results are described. A discussion of the results is given in the last section.

## 2. Methods

In this section, a method for accurately estimating the angle of the lower-limb joint even with varying walking speed is described. (a) For angle estimation, weights were learned from IMU data using an artificial neural network (the input value is IMU data, the output value is joint angle). Since the experiment was conducted on the four-step walking speed, four sets of weights were stored in the MCU. (b) Similarly, IMU data were trained to predict walking speed (the input value is IMU data, the output value is walking speed). Therefore, the procedure for estimating the angle was as follows. First, the walking speed was predicted using the weight obtained in (b). Second, the angle was estimated by selecting the correct weight from the weights obtained in (a) according to the predicted walking speed.

### 2.1. Exoskeleton Robot Design and Walking Data

**Figure 1:**
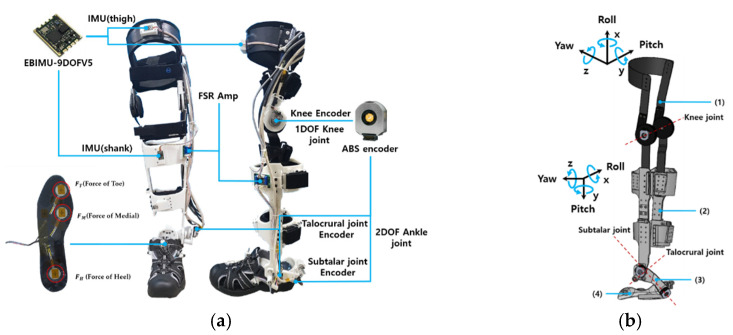
Structure of the developed exoskeleton robot: (**a**) Inertial measurement unit (IMU), force-sensitive resistors, absolute encoder, and force-sensitive resistors amplifier. (**b**) 3D-modeled lower limb exoskeleton robot, inertial measurement unit, axis of Inertial meas-urement units (IMU), knee, and ankle joint position. (1) Knee orthosis (thigh), (2) frame for tibia (shank), (3) frame for talus, (4) frames for calcaneus.

The designed exoskeleton robot is a lower-limb exoskeleton robot developed, as shown in Figure 1, that is driven by a bi-directional tendon-driven actuator [25]. This exoskeleton robot consists of 1 degree of freedom (DoF) for the knee and 2 DoFs for the ankle. Straps were used to fix the shank and thigh. The knee and ankle are equipped with a 12-bit absolute encoder (ABS encoder) that can measure the angle of rotation during walking from 0° to 360°. 

IMU sensors were also attached to the shank and thigh to collect data from the gyroscope and accelerometer while walking. The knee joint can be moved 180° considering the range of motion of the human knee. Likewise, the talocrural joint of the ankle can move 90°, and the subtalar joint can move 150°. To estimate the gait section with the IMU sensor, a foot sensor with three FSR sensors that can determine the gait section was mounted on the sole. The mass of the exoskeleton robot can be found in Table 1. We used an AMT 203-V (Absolute) encoder (CUI Inc., Gyeonggi-do, Korea) and an EBIMU-9DOFV5 IMU (E2BOX Inc., Shanghai, China).

**Figure 2:**
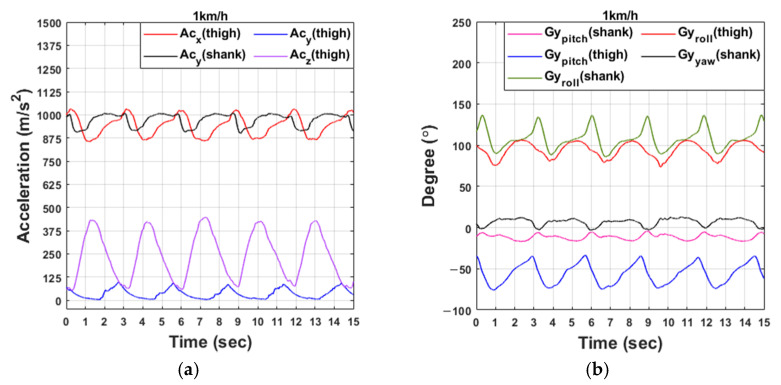

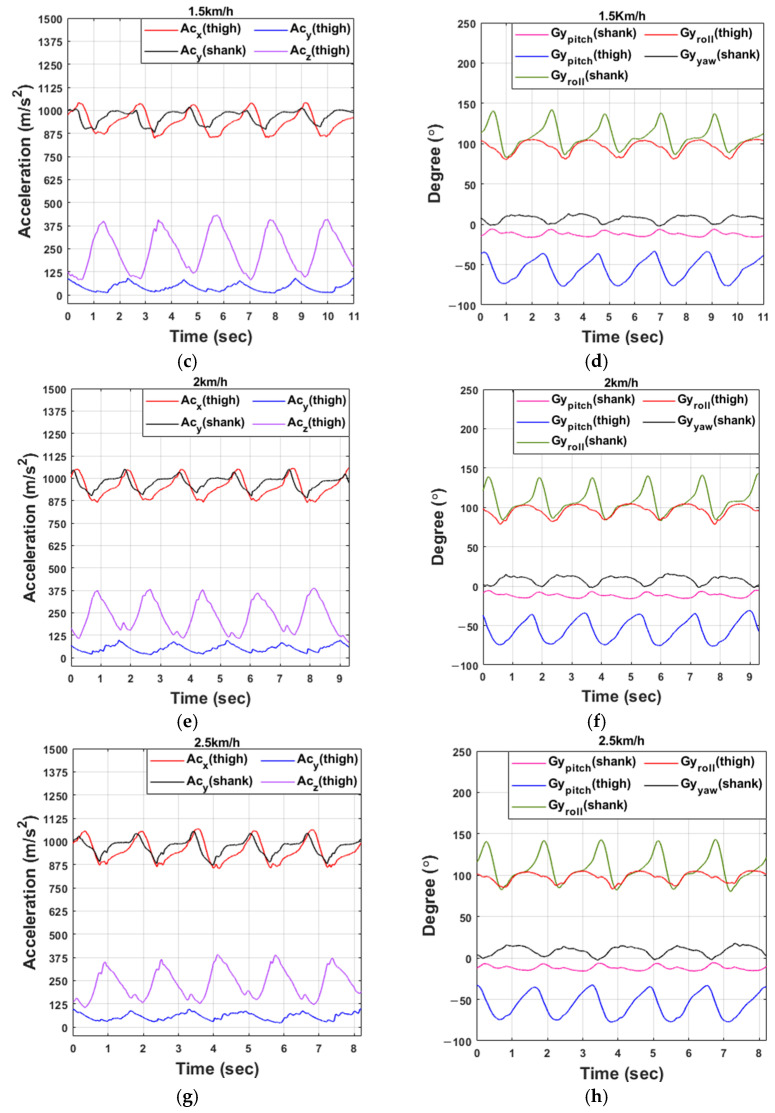
IMU sensor data obtained by walking on a treadmill: (**a**,**c**,**e**,**g**); accelerometer data: (**b**,**d**,**f**,**h**) gyroscope data. (From top to bottom, 1, 1.5, 2, and 2.5 km/h).

Figure 2 shows IMU sensor data obtained by walking on a treadmill. IMU data were acquired every 1 microsecond. The acceleration value in Figure 2 is the acceleration value multiplied by 1000. Four suitable walking speeds for gait training were selected: 1, 1.5, 2, and 2.5 km/h. As shown in Table 2, the range standard deviation (SD) of the accelerometer was more than 7, but that of the gyroscope was less than 3.3. Therefore, as the walking speed increased, the gait cycle period decreased; however, there was little difference in the range of gyroscope data. On the other hand, the accelerometer data had a change in amplitude according to the walking speed. As the walking speed increased, the rising peaks of $Ac_{x}\left(\mathrm{thigh}\right)$ and $Ac_{y}\left(\mathrm{shank}\right)$ increased, and the descending peak of $Ac_{z}\left(\mathrm{thigh}\right)$ increased. Therefore, the gyroscope data were used to predict the walking speed due to the change of period and constant amplitude. 

### 2.2. Gait Phase Analysis

**Figure 3:**
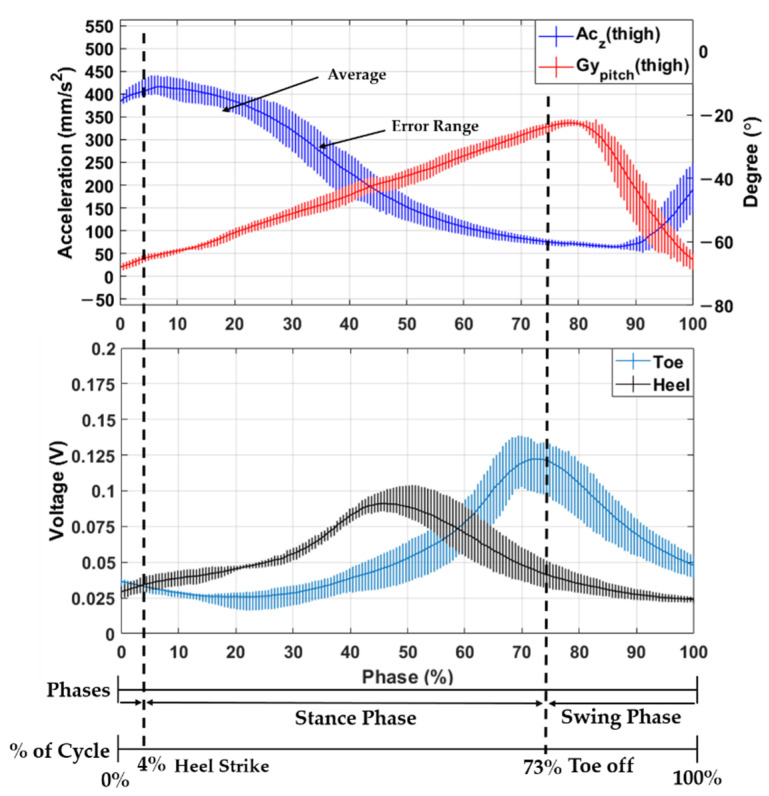
The average and error range of data from the IMU sensor and foot sensor during 50 gait cycles.

Walking is typically divided into a stance phase and a swing phase. When the foot is on the ground, this is called the stance stage, and when the foot is off the ground, this is called the swing stage. The stance phase and swing phase can also be divided into heel strike and toe-off points as shown in Figure 3. The walking section can be determined through the foot sensor data received during walking. The toe data of the foot sensor are the average values of the sum of the data of $F_{T}$ and $F_{M}$ shown in Figure 1a, and the heel data are the data of $F_{H}$. 

At the heel strike point, the z-direction accelerometer value attached to the thigh decreases from the highest point of the rising peak, and at the toe-off point, the pitch direction gyroscope value attached to the thigh decreases from the highest point of the ascending peak. Therefore, since the gait section can be estimated through the accelerometer data in the z-direction of the IMU sensor and the gyroscope data in the pitch direction, it is possible to estimate the gait section instead of the foot sensor even for patients who cannot produce accurate pressure on the ground.

### 2.3. Weight Analysis Using Neural Network

**Figure 4:**
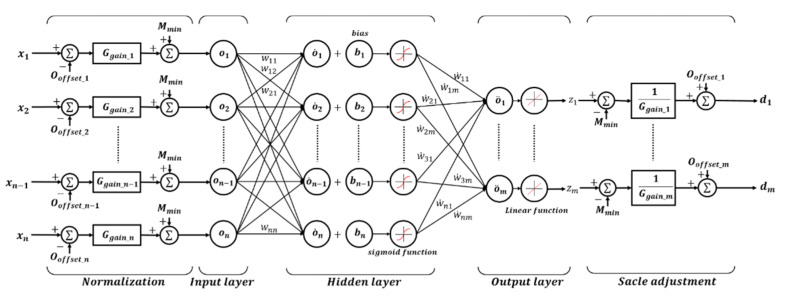
The structure of an artificial neural network. Both the walking speed estimation and the angle estimation of each joint are composed of neural network algorithms of the same structure.

Three target values were estimated: knee angle, ankle angle, and walking speed. All are determined by the same neural network algorithm, as shown in Figure 4. The neural network for the knee consists of 5 input neurons (n=5), 10 hidden neurons, and 1 output neuron (m=1) as an estimate of the angle of the knee joint. The neural network for the ankle consists of 9 input neurons (n=9), 10 hidden neurons, and 2 output neurons (m=2) as an estimate of the angle of the talocrural joint and subtalar joint of the ankle joint. The neural network algorithm for gait speed estimation consists of 5 input neurons (n=5), 10 hidden neurons, and 1 output neuron (m=1). 

The exact input neurons for each target are shown in Table 3. The input data were converted between −1 and 1 through normalization before entering the input layer. In addition, the data from the output layer were converted to the original scale through the scale adjustment process and matched with the actually measured knee and ankle angles. The sigmoid function of the hidden layer was selected as in Equation (1) as a symmetric transfer function, where v is the sum of the bias values after multiplying the input data and the weight. The backpropagation algorithm uses the Levenberg–Marquardt method, which is Equation (2). The number of hidden neurons and the back propagation algorithm were selected in consideration of the smallest estimation error and the amount of computation through repeated experiments.

Among the walking data obtained by taking a total of 50 steps on the treadmill, the rotation angles of the knee and ankle were selected as the target for the 25 step data, and the weight was updated through the backpropagation algorithm to calculate the appropriate weight. Of the data samples, 70% were used to train the weights, and 15% were not used for training but were used to measure the performance of the neural network during or after training. The remaining 15% were used to stop learning by measuring the generalization of the neural network. The number of epochs for weight learning was 41 for 1 km/h, 43 for 1.5 km/h, 61 for 2 km/h, and 98 for 2.5 km/h. As the walking speed increased, the amount of learning increased.

(1) $$sigmoid=\frac{2}{1+e^{-2v}}-1$$

(2) $$m\left(t+1\right)=m\left(t\right)-{\left[J^{T}J+\mu I\right]}^{-1}J^{T}E.$$

Here, m is the weight between each layer. J is a jacobian matrix containing the first derivative of the neural network error for weights and biases, and E is a vector composed of neural network errors. The scalar μ changes in size according to the error reduction rate and becomes similar to the Gauss–Newton method as it decreases, and the convergence speed increases. As it increases, it becomes similar to the gradient descent, and the convergence speed decreases.

### 2.4. Angle Estimation Simulation

**Figure 5:**
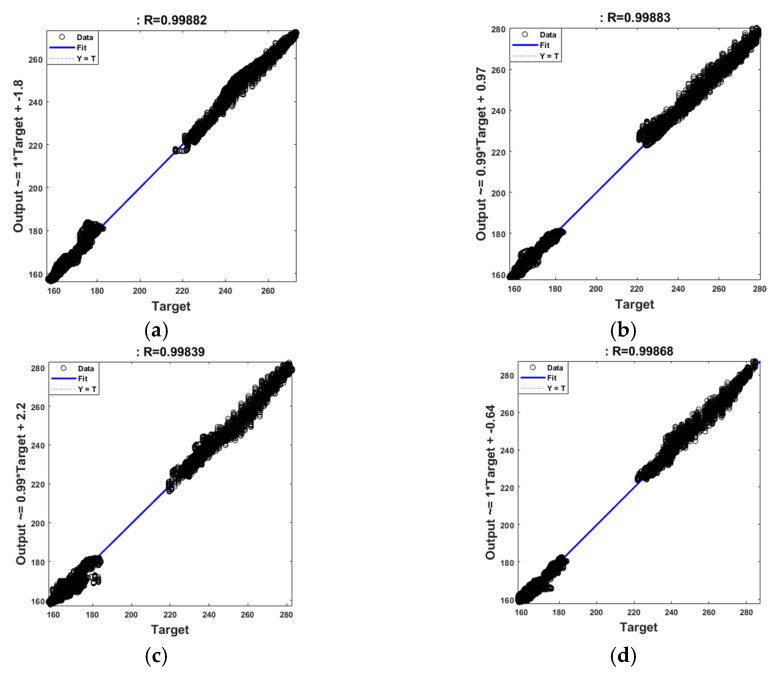
The performance of the neural network was verified with a regression plot: (**a**) walking speed at 1 km/h; (**b**) 1.5 km/h; (**c**) 2 km/h; and (**d**) 2.5 km/h.

In the angle estimation simulation, the weights and bias values were learned from 25 steps of the total walking data of 50 steps, and a feedforward neural network was conducted based on the IMU data for the remaining 25 steps to test the learned weights. As a result of the simulation, the mean absolute error (MAE) and standard deviation (SD) are shown in Table 4. The MAE of all joints tended to show a larger error as the walking speed increased. In the case of the knee, the angle change during walking was large, and the MAE tended to be larger than the ankle joint. Figure 5 shows the R (regression) value that confirms the performance of the neural network. For all walking speeds, the R-value is located close to a 45° straight line. Therefore, all of the R values were above 0.998, showing excellent neural network performance.

### 2.5. Knee and Ankle Angle Estimation Algorithm

**Figure 6:**
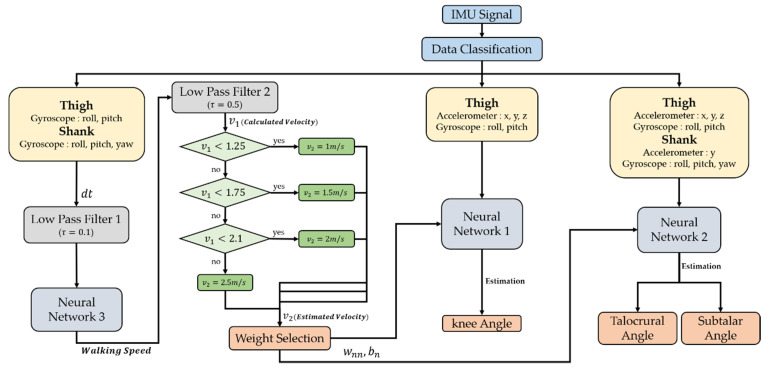
Schematic block diagram showing the sequence of algorithms for estimating knee and ankle angles according to walking speed. The contents of the neural network block are shown in Figure 4.

Figure 6 is a schematic diagram of knee and ankle angle estimation including a feedforward neural network. A total of 12 accelerometers and gyroscope data from the IMU sensor attached to the thigh and shank were used to classify the necessary data through data classification to estimate the walking speed and angle of each joint. In this process, the knee is classified into five inputs, the ankle into nine, and the walking speed into five datapoints. Only gyroscope data were used to estimate the walking speed. Five types of gyroscope data were used, excluding data in the yaw direction on the IMU attached to the thigh as shown in Figure 6. 

To obtain data related to the walking speed value, the gyroscope data were differentiated, and the data were organized with a low pass filter (τ=0.1). The organized data were calculated through the neural network as shown in Figure 4. The output data of the neural network were again organized through a low pass filter (τ=0.5), and a walking speed value equal to the speed of the treadmill was derived through a logic process. The accuracy of the angle estimation of each joint was increased by selecting an appropriate weight and bias value according to the walking speed and using this as a weight when entering the neural network for estimating the knee and ankle angles. The time constant of the low pass filter used for accelerometer data was selected as the constant with the smallest estimation error through repeated experiments.

(3) $$\dot{a}\left(t\right)=\frac{\tau \dot{a}\left(t-1\right)+t_{I}a\left(t\right)}{\tau +t_{I}}$$

Equation (3) is the applied low pass filter. Here, $\dot{a}$ is accelerometer data to which a low pass filter is applied, and τ is a time constant. a is the accelerometer data before passing through the low pass filter. The data acquisition time in MCU is 1 microsecond. Thus, $t_{I}$ is 0.001.

## 3. Experimental Setting and Results

**Figure 7:**
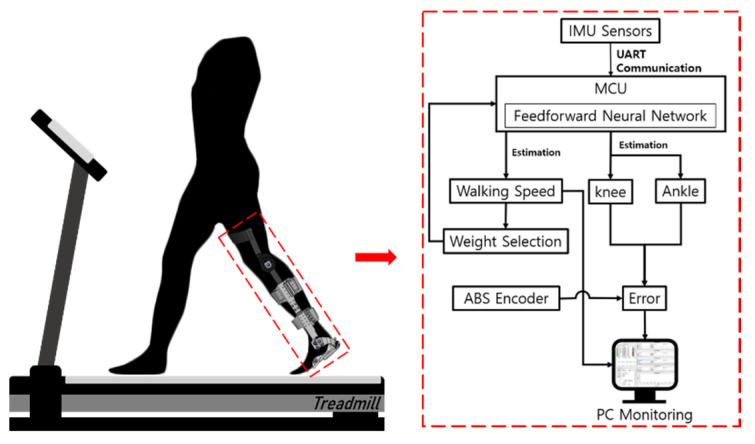
Schematic diagram of the experimental setup for estimation of the joint angle and walking speed.

As shown in Figure 7, an experiment was conducted on a treadmill to verify the validity of the method of estimating the wearer′s joint angles during walking in real-time. The experiment was divided into three parts. First, the joint angle was estimated when the walking speed was constant at 1, 1.5, 2, and 2.5 km/h. Second, an experiment was conducted to estimate the angle at a speed varying from 1 to 2.5 km/h. Third, a free walking experiment was conducted to confirm the angle estimation for general walking on the ground. The constant gait experiment was conducted by selecting a weight appropriate for each speed, and the variable gait experiment was conducted by selecting an appropriate weight through estimating walking speed. 

**Figure 8:**
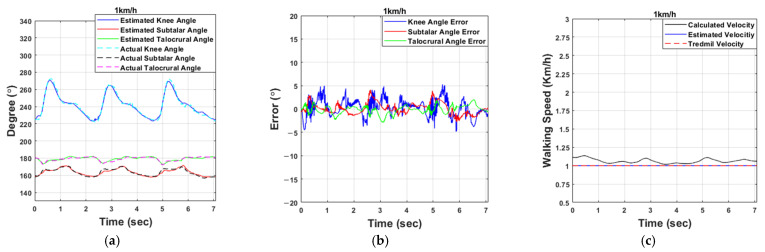

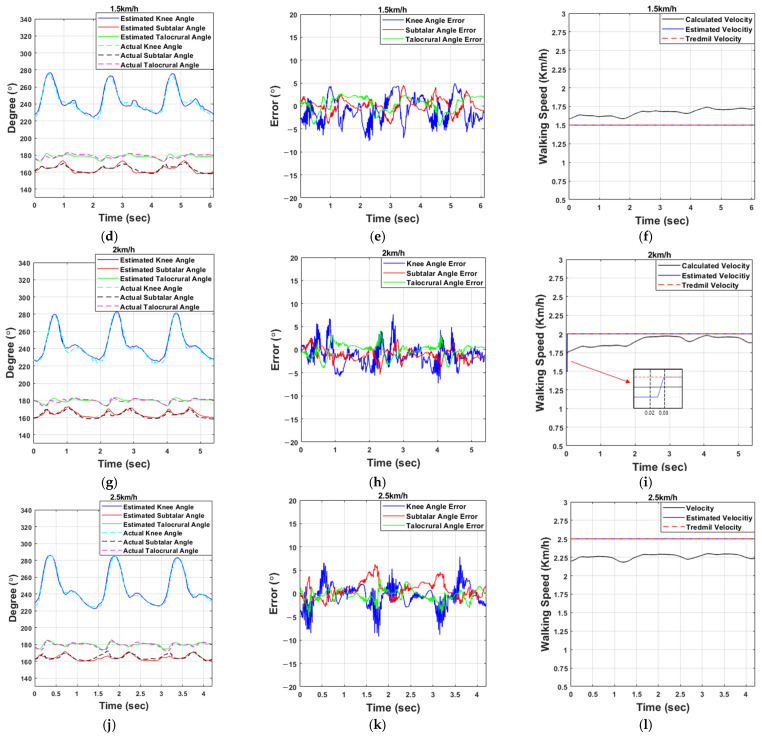
Experimental results for estimating the knee and ankle joint angles: (**a**,**d**,**g**,**j**) Results of estimating the angles of the knee and ankle. (**b**,**e**,**h**,**k**) Results of the angle estimation errors. (**c**,**f**,**i**,**l**) Results of the walking speed estimations (from top to bottom, 1, 1.5, 2, and 2.5 km/h).

As shown in Table 5, the interrupt time of the microcontroller unit (MCU) was one millisecond. For the first experiment, the results are shown in Figure 8. 

**Figure 9:**
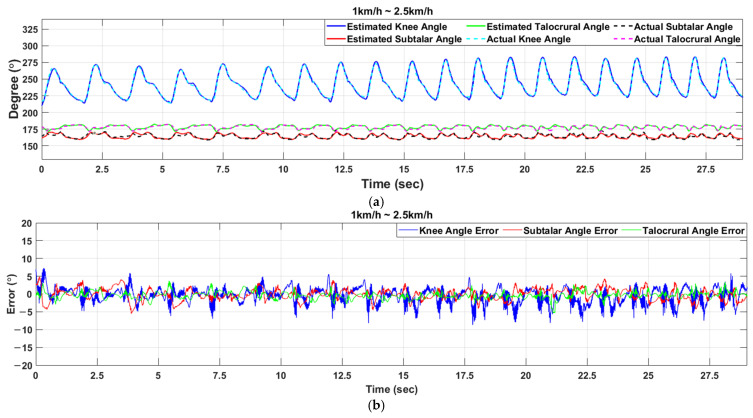

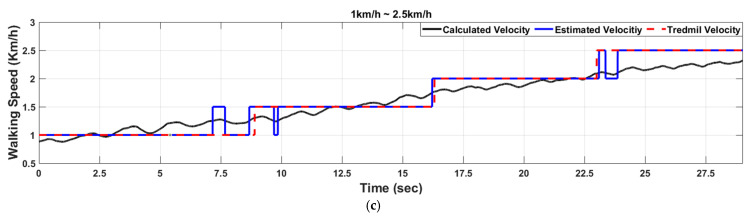
Experimental results for the estimation of knee and ankle angle during walking speed increases: (**a**) Results of estimating the angle of knee and ankle; (**b**) Results of the angle estimation error; (**c**) The result of walking speed estimation.

At a walking speed of 1 km/h, the knee angle estimation error was within ±5°, and the maximum error was 5°; at 1.5 km/h the maximum error was 7.64°; at 2 km/h it was 7.66°; and at 2.5 km/h it was 9.26°. The angle estimation error of the ankle joint was mostly within ±5%, showing a mostly stable appearance. The subtalar joint is a joint that balances the gait. As the walking speed increases, the gait stability decreases. Due to the pattern change, the subtalar angle estimation error increases as the walking speed increases. The estimation of the walking speed showed results that were mostly consistent with the speed of the treadmill. Table 6 shows the mean absolute error (MAE) and standard deviation (SD) of the error according to the walking speed. Table 7 shows the percent error for angle estimation. The percent error for estimating the angle of the knee was about 6% but about 3% for the ankle. Table 8 shows the initial position of each joint and the range of motion (RoM) of the joint during walking. The subtalar joint moved at a constant RoM even when the walking speed increased, but the knee and talocrural joint RoM increased. The second experiment came out as shown in Figure 9, and the walking speed was increased every five steps. 

Even when the walking speed increased, the joint angle was estimated close to the desired joint angle. Table 9 shows the mean absolute error (MAE) and standard deviation (SD) of the error with increasing walking speed. As shown in Figure 9b, The angle estimation error increased from 7.2° to 8.7° in the knee joint, from 4.5° to 5.5° in the subtalar joint, and from 3.7° to 5.3° in the talocrural joint as the walking speed increased. However, the overall MAE was less than 1.7°, and the joint angle was well estimated. As shown in Figure 9c, estimating the walking speed confirmed that the speed changed with each section when the walking speed increased. Table 10 shows the percent error for angle estimation. Similar to the experiment where the walking speed was constant, the percent error for the knee angle estimation was about 6%, and the ankle was about 3%. The walking speed estimation accuracy was 94.5%, and this was well estimated even in experiments where the walking speed changed. 

**Figure 10:**
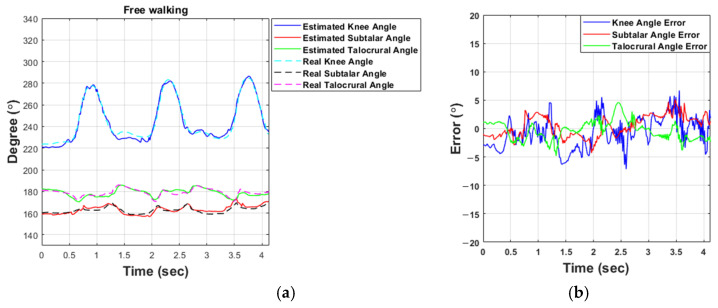
Experimental results for the estimation of the knee and ankle angles when free walking: (**a**) Results of estimating the angles of the knee and ankle. (**b**) Results of the angle estimation error.

Figure 10 shows the results of a free walking experiment on the ground, and the error in the free walking experiment is about 5%. As can be seen in Table 11 and Table 12, the MAE for the knee is 1.69°, the talocrural joint is 0.82°, and the subtalar joint is 1.29°. However, compared to the experiment of the treadmill, it can be seen that the MAE and SD have increased. Overall, the error slightly increased, but it can be seen that the angle estimation had a good performance in the free walking experiment.

## 4. Discussion

In this study, we propose an angle estimation algorithm using a neural network to control the angle of the joint of the lower-limb exoskeleton robot, which was developed to assist walking. The joints to estimate the angles are the talocrural joint and the subtalar joint, which include 2 DoFs for the ankle and the knee. The walking speed of the joint angle estimation experiment was 1–2.5 km/h, which was set for the speed of patients who require walking training. The experiment was divided into three cases: a constant walking speed for general gait training, a case in which the walking speed was changed to adapt to the changes in walking speed, and free walking to determine the angle estimation on the ground.

The neural network algorithm trained weights and bias values using the programmed neural network algorithm in MATLAB software. The proposed algorithm predicted the walking speed and delivered an appropriate weight according to the predicted walking speed to estimate the angle of the knee and ankle in real-time. For both the knee and ankle angle estimation and gait speed prediction, the weights and bias values learned by the neural network were used in advance, and the feedforward neural network was calculated by the MCU to estimate the walking speed and the angle of the joint. The walking speed prediction used gyroscope data from the IMU sensor attached to the exoskeleton robot, and both the accelerometer and gyroscope data were used to estimate the angle of the knee and ankle.

In the experiment at a constant walking speed, the real-time angle estimation error was relatively larger than that in the simulation; however, the proposed algorithm with a MAE within 2.5° estimated the wearer′s intention well in the experiment in which the walking speed changed, the MAE was accurately estimated to be less than 1.7° as a result of using an appropriate weight. In addition, the free walking experiment on the ground also estimated the MAE to be less than 2.4°. Patients who have problems with proper functioning, such as lower extremity patients, have difficulty in estimating joint angles with sensors, such as torque and EMG, and thus the proposed algorithm has high expectations for gait rehabilitation.

In addition, the subtalar joint plays an important role in balancing the gait and is one of the factors to be considered in gait rehabilitation. Therefore, unlike other joints, even when the walking speed increases, the RoM is almost constant. However, most studies on angular estimation are conducted only for talocrural joints. Therefore, estimation of the walking angle of the subtalar joint is expected to be an advantage of rehabilitation in the future. In addition, unstable gait data were also estimated by estimating the angle of the joint in real-time. Therefore, more stable and accurate angle estimation is expected to be possible if the proposed method is performed with only stable walking data by measuring the stability of walking.

In conclusion, this study proposes a method of estimating the continuous angle of the joint even when the walking speed changes using an artificial neural network, unlike previous studies that simply estimate the gait section using IMU data. It was proved through experiments that the importance and angle of the subtalar joint can be estimated. However, the proposed method is not accurate enough to estimate the walking intentions of everyday life.

Therefore, in addition to artificial neural networks (ANNs), performance can be improved through deep learning with a large number of hidden layers and convolutional neural networks (CNNs), which are image processing techniques. This method can estimate not only simple gait training but also various walking intentions of daily life. In addition, the improved system can be used not only for rehabilitation robots but also for industrial and military robots.

## Figures and Tables

**Table 1:** Mass analysis of the manufactured exoskeleton robot.

Part	Volume	Weight (kg)
Frames (2), (3) and (4)	1	0.72
Knee orthosis (1)	1	0.7
Bowden cable, encoder and IMU	7	0.58
Shoe	1	0.35
Total	N/A	2.35

**Table 2:** Accelerometer and gyroscope data according to the walking speed.

IMU	Unit	1 km/h	1.5 km/h	2 km/h	2.5 km/h	Range SD
${\mathrm{Ac}}_{x}\;\left(\mathrm{thigh}\right)$	$mm/s^{2}$	855.77–1031.93	849.14–1041.47	864.5–1055.81	853.84–1067.27	14.86
${\mathrm{Ac}}_{y}\;\left(\mathrm{shank}\right)$		901.02–1009.54	879.28–1017.68	890.52~1050.5	872.04–1054.47	7.55
${\mathrm{Ac}}_{y}\;\left(\mathrm{thigh}\right)$		3.32–98.55	10.13–92.54	16.56–96.71	22.44–97.5	16.57
${\mathrm{Ac}}_{z}\;\left(\mathrm{thigh}\right)$		51.69–447.55	81.81–432.17	85.17–387.42	104.1–389.31	15.82
Pitch (shank)	degree	−16.77–−4.3	−16.15–−5.95	−15.94–−5.2	−15.72–−5.62	1.93
Pitch (thigh)		−75.7–−33.75	−76.69–−33.46	−75.94–−31.1	−77.25–−32.62	3.29
Roll (shank)		85.99–136.6	83.11–142	83.39–142.97	80.52–143.12	1.43
Roll (thigh)		73.69–106.13	80.51–105.22	78.63–104.68	83.58–105.21	1.23
Yaw (shank)		−3.01–12.89	−1.98–13.16	−1.56–15.83	−2.55–17.65	0.41

**Table 3:** Input neurons for estimation of the knee, ankle, and walking speed.

Target	IMU Position	IMU Data	Input Neurons
Knee	thigh	accelerometer	x, y, z
		gyroscope	roll, pitch
Ankle	thigh	accelerometer	x, y, z
		gyroscope	roll, pitch
	shank	accelerometer	y
		gyroscope	roll, pitch, yaw
Walking Speed	thigh	gyroscope	roll, pitch
	shank	gyroscope	roll, pitch, yaw

**Table 4:** The mean absolute error (MAE) and standard deviation (SD) of the angle estimation simulation according to walking speed.

Joint	1 km/h		1.5 km/h		2 km/h		2.5 km/h	
	MAE	SD	MAE	SD	MAE	SD	MAE	SD
Knee joint	0.75	0.56	0.91	0.77	1.02	0.88	1.07	0.92
Subtalar joint	0.58	0.48	0.68	0.56	0.95	0.78	0.82	0.76
Talocrural joint	0.47	0.39	0.55	0.51	0.61	0.51	0.66	0.55

**Table 5:** Parameters of the experimental setup.

Component	Parameter	Specification
Motor	Input voltage	24 V
	Watts	103 W
	Speed limit	3200 RPM
	Gear ratio	100:1
MCU	System clock	160 MHz
	Interrupt time	1 microsecond

**Table 6:** The mean absolute error (MAE) and standard deviation (SD) of real-time angle estimation according to the walking speed.

Joint	1 km/h		1.5 km/h		2 km/h		2.5 km/h	
	MAE	SD	MAE	SD	MAE	SD	MAE	SD
Knee joint	1.48	1.11	2.46	1.56	2.06	1.54	1.81	1.64
Subtalar joint	0.97	0.78	1.17	1.04	1.37	0.88	1.43	1.23
Talocrural joint	0.71	0.58	1.55	0.82	2.08	1.04	1.68	1.01

**Table 7:** Percent error (PE) of angle estimation simulation according to walking speed.

Joint	1 km/h	1.5 km/h	2 km/h	2.5 km/h
	Percent Error			
Knee joint	4.71%	5.27%	5.07%	5.25%
Subtalar joint	2.01%	2.19%	2.79%	3.07%
Talocrural joint	1.47%	2.73%	2.37%	3.25%

**Table 8:** Initial position and range of motion (RoM) of each joint.

Joint	Initial Position	1 km/h	1.5 km/h	2 km/h	2.5 km/h
		Maximum, Minimum (RoM)			
Knee joint	216	272, 222 (50)	274, 221 (53)	281, 223 (58)	285, 224 (61)
Subtalar joint	168	170, 156 (14)	171, 158 (13)	171, 158 (13)	171, 159 (12)
Talocrural joint	180	181, 172 (9)	182, 172 (10)	183, 173 (10)	186, 172 (14)

**Table 9:** The mean absolute error (MAE) and standard deviation (SD) of the angle estimation error according to the walking speed change.

Joint	1–2.5 km/h	
	MAE	SD
Knee joint	1.69	1.43
Subtalar joint	1.29	1.01
Talocrural joint	0.82	0.69

**Table 10:** Percent error (PE) of angle estimation simulation according to walking speed.

Joint	1 km/h~2.5 km/h
	Percent Error (PE)
Knee joint	4.93%
Subtalar joint	3.08%
Talocrural joint	2.41%

**Table 11:** The mean absolute error (MAE) and standard deviation (SD) of the angle estimation error according to the free walking.

Joint	Free Walking	
	MAE	SD
Knee joint	2.37	1.62
Subtalar joint	1.69	0.97
Talocrural joint	1.37	1.03

**Table 12:** Percent error (PE) of angle estimation simulation according to free walking.

Joint	Free Walking
	Percent Error (PE)
Knee joint	5.33%
Subtalar joint	3.22%
Talocrural joint	3.04%

## Data Availability

Data are contained within the article.
